# Body Weight, Obesity Perception, and Actions to Achieve Desired Weight among Rural and Urban Ghanaian Adults

**DOI:** 10.1155/2020/7103251

**Published:** 2020-03-13

**Authors:** Nana Ama Frimpomaa Agyapong, Reginald Adjetey Annan, Charles Apprey, Linda Nana Esi Aduku

**Affiliations:** Department of Biochemistry and Biotechnology, Kwame Nkrumah University of Science and Technology, Kumasi, Ghana

## Abstract

**Background:**

Accurate body weight perception is important to maintaining an ideal body weight. In Africa, a preference for a larger body size and its association with health and wellbeing has been well documented. It remains speculative if these perceptions have changed or improved and if differences exist among rural and urban dwellers. The main aim of this study was to assess the body weight and obesity perceptions among rural and urban Ghanaians.

**Methods:**

This cross-sectional study involved 565 participants. The Stunkard figure rating scale was used to assess the body weight perception of participants. Participants were to choose from the scale figures they perceived to represent their current body weight, desired body weight, ideal body weight, ideal look for a wealthy person, ideal look for a woman with children, and ideal look for a woman without children. Additionally, participants were asked to describe obesity and its threat to health in their terms. Responses of participants to the above questions are presented as frequencies. Differences between rural and urban participants as well as males and females with respect to the median figure chosen for each question were determined by Mann–Whitney *U* test.

**Results:**

The median age of participants was 40 (IQR 26). The prevalence of overweight and obesity observed among participants was 52.8%. The most frequently selected figure as current body image was figure 5 (23.5%). Figure 4 was most frequently chosen by both males (37.2%) and females (24.6%) as their desired body image (27.4%). Male participants (41.8%) chose figure 5 as ideal for their gender while females (27.4%) maintained figure 4 as ideal for their gender. Study participants associated overweight with wealth and childbirth, and attributed their current weights to hereditary (27%) and childbirth (27%). Most participants were not taking steps to achieve their desired body image, and only a few engaged in both dieting and exercise to lose weight. Majority of participants described obesity as the accumulation of fat (91.0%) and viewed it as a threat to health (91.0%). Differences were observed among rural and urban participants with regard to the figure chosen as ideal for a wealthy person.

**Conclusion:**

Results from this study show an improvement in obesity perception and the acknowledgment of obesity as a threat to health. There was a desire for a normal-weight figure among study participants. Attribution of current body weight to hereditary and childbirth seems to be a hindrance to the implementation of actions to achieve this normal figure weight. Public health education, screening for overweight and obesity, creation of supportive food environments, and culture-sensitive interventions are promising to curbing the obesity menace.

## 1. Introduction

The rise in obesity rate and prevalence is an interplay of many factors: socioeconomic status, marital status, lifestyle factors, poverty, and the food environment [1]. Several policies and actions have been proposed as measures to curb the obesity menace. These have involved regulation of the food environment mostly around schools, behavior change communication and regulation of specific ingredients especially fat and sugar in foods [2–4]. Within the African context, preference for a heavier body size and its link to wealth and wellbeing may render efforts aimed at resolving obesity through a healthful food environment futile [1, 5]. Behavior change communication also works best when there is a correct perception of body size and appreciation of the health risk of obesity. Body weight perception influences eating behaviors and patterns as well as the will to reduce or maintain weight [6, 7]. Culturally entrenched perceptions of weight for both women and men in Africa drive a preference for overweight and obesity; meanwhile, obesity is associated with all-cause mortality and reduced quality of life among populations [8–10].

The nutrition transition is characterised by high intake of calorie-dense foods and low levels of physical activities. It is driven by environmental factors such as abundance of ultra-processed foods and motorisation of transport [11]. This undoubtedly is one of the main drivers of the obesity pandemic. However, these environmental factors alone do not explain the prevalence and incidence of obesity in Africa. Food environments in developed continents are more obesogenic compared to developing continents like Africa, but obesity prevalence in Africa continues to rise [12]. Africa is still struggling with a high burden of undernutrition among children, and infectious diseases like HIV/AIDS and tuberculosis [13]. Amidst these health challenges, body weight within the overweight and obese categories are seen as the absence of the aforementioned conditions. Staple and whole foods which are healthier food options compared to processed calorie-dense foods may be overconsumed to promote weight gain. Therefore, the presence of an obesogenic food environment may just enhance rather than be the cause of obesity among this population. For instance, among African immigrants, Renzaho et al. found that overweight and obesity were associated with beauty and wealth. Parents, therefore, restricted their childrens' involvement in physical activity in order to promote weight gain [14]. A systematic review by Toselli et al. revealed that African residents generally preferred a larger body size compared to African immigrants who tend to internalize the western cultural ideal of slimness [15].

Studies have been conducted to assess the perceptions of body weight among Africans. Most of these studies have focused on adolescents, women and urban settlers. A systematic review conducted by Agyemang et al. reported diverse views on obesity and body weight perceptions in sub-Saharan Africa. While earlier studies in the review documented preference for an obese or overweight body figure, latter studies found that most obese participants desired to lose weight and associated obesity with risk of developing chronic diseases [1]. Research by Okop et al. on the other hand revealed that overweight individuals did not perceive obesity as a threat to health and were less likely to want to lose weight as compared to individuals who were obese [16]. A higher proportion of overweight and obese participants tend to be satisfied with their weight and express willingness to lose weight with a perceived cardiovascular disease risk [17]. In South Africa, women perceived overweight and obesity as threats to health but preferred to be obese or overweight because of the fear of being stigmatized as HIV positive [18].

In Ghana, Benkeser et al. reported that overweight and obese women desired to lose weight, contrarily [19]. Mogre et al. documented a distorted view of obesity among civil servants and underestimation of actual body weight among the study participants [20]. A latter report by Appiah et al. established that urban Ghanaian women preferred a larger body size and associated overweight with wealth [5]. It remains unknown if these perceptions have changed or improved. Prevalence of obesity is higher among urban dwellers, but no study has assessed the difference in perceptions along community lines. The main aim of this cross-sectional study was to assess the differences in perception of body weight among rural and urban Ghanaian adults.

## 2. Methods

**Figure 1:**
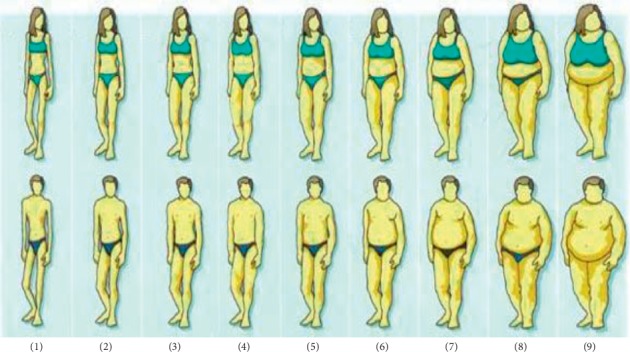
Stunkard figure rating scale [22].

The study was a cross-sectional community-based study. The body mass index (BMI) of all participants was determined from weight and height measurements (weight in kilograms/height in meters^2^). The Stunkard figure rating scale was used to assess the body weight perception of study participants (Figure 1). The scale is validated and has been used by many researchers to assess body weight perceptions and produces similar results as other African adopted scales [21]. The scale consists of nine figures with established BMIs ranging from severely wasted to morbidly obese [22]. Participants were asked to choose from the scale figures that represented their current body image (CBI), desired body image (DBI), ideal image for their gender, ideal look for the opposite sex, wealthy man, wealthy woman, woman with children, and a woman without children. Feel minus Ideal Discrepancy (FID) was determined by subtracting the CBI of participants from their DBI. Additionally, participants were asked the reason for their current weight, how they will describe obesity, and whether they viewed obesity as a threat to health or not. Participants were also asked to indicate the actions and steps they were taking to achieve their desired weight.

### 2.1. Study Site

The study was conducted in an urban (Ahodwo) and a rural (Ejuratia) community in the Ashanti region of Ghana.

### 2.2. Sample Size and Sampling

A total of 565 adults (18 years and above) 292 from rural and 272 from urban were involved in this study. By gender, 113 males and 452 females participated in this study. This was a community-based study conducted within households. A hosuehold refers to individuals who feed from the same pot. For each community, a central point was determined and the community stratified into sections starting from the central point. Systematic sampling was applied and used to select households on a 1 in 5 ratio for the urban community (Ahodwo) and 1 in 3 ratio for the rural community (Ejuratia) . Within each household, one person was recruited to be part of the study. In households where there were both a male and a female who qualified, the male was selected to ensure gender balance.

### 2.3. Ethical Clearance

The Council for Scientific and Industrial Research (CSIR) issued ethical clearance for the study (RPN 011/CSIR-IRB/2017). Local government officials approved for the research to be carried out within the selected communities, and participants signed an informed consent to indicate willingness to participate.

### 2.4. Statistical Analysis

Statistical Package for Social Sciences (IBM SPSS) version 23 was used to analyze the data. BMI is reported as median because the data were not normally distributed. Responses for each figure are presented as frequencies and percentages. Differences between rural and urban participants and males and females with regard to mean figures chosen was determined by the Mann–Whitney *U* test.

## 3. Results

**Figure 2:**
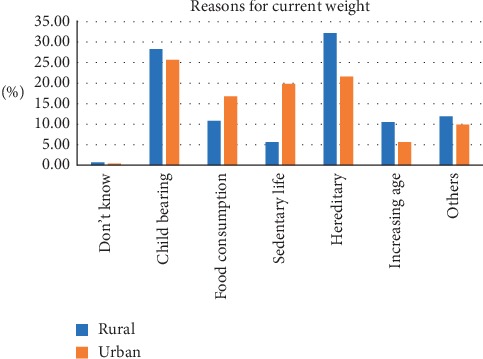
Reasons for current weight.

**Figure 3:**
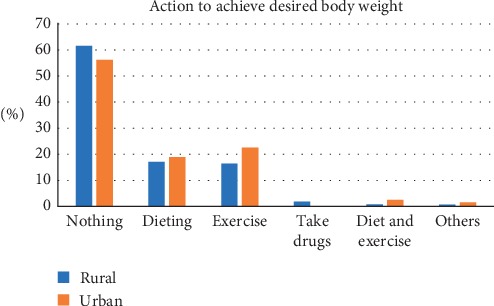
Action to achieve desired body weight.

**Figure 4:**
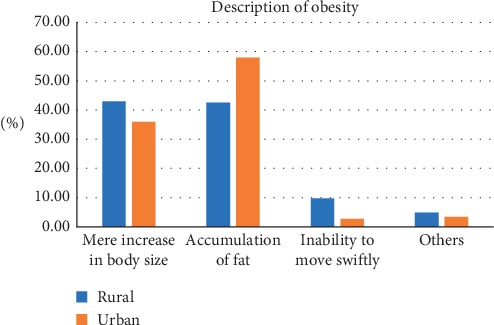
Description of obesity.

**Figure 5:**
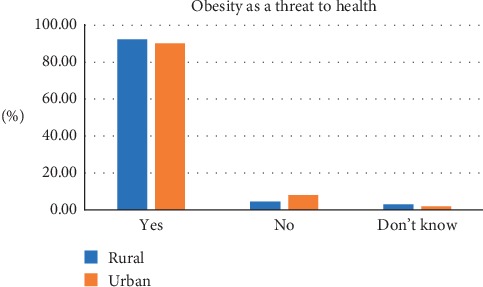
View of obesity as a threat to health.

The median age of participants was 40 (IQR 26). The most frequently chosen figure as CBI was figure 5 for both males and females. The corresponding measured median BMI was 26.0 (IQR 5.8) for rural and 26.8 (IQR 5.9) for urban. By gender, the corresponding measured median BMI was 23.2 (2.9) for males and 27.7 (5.1) for females. The least selected CBI was figure 9 and the corresponding BMI was 42.4 (0) for rural and 55.5 (0) for urban.  Table 1 shows the current body image perceptions of the participants. Most participants selected figure 4 as their desired body image and this was the case for both males (37.2%) and females (24.6%). The figure most frequently selected as unhealthiest was figure 9. Majority of men selected figure 4 (41.6%) as ideal for a woman while figure 5 (35%) was frequently selected by females as ideal for a man. Most of the participants selected figure 3 as ideal for a woman without children while figure 5 was chosen as the ideal for a woman with children no longer reproducing. Tables 2 and 3 show the body weight perceptions of participants. Significant differences were observed between rural and urban participants with respect to the median image of a wealthy man (rural 6 (IQR 3); urban 6 (IQR 2); *p* value <0.001), a wealthy woman (rural 6 (IQR 2); urban 5 (IQR 2); *p* value <0.001), and the unhealthiest (rural 9 (IQR 1); urban 9 (IQR 0.25); *p* = 0.021) Significant differences were observed between males and females with respect to figure chosen as desired body image (male 4 (IQR 1); female 4 (IQR 2); *p* value <0.001), ideal for gender (male 4 (IQR 2); female 4 (IQR 1); *p* value 0.008), and ideal for the opposite sex (male 4 (IQR 2); female 5 (IQR 1); *p* value <0.001) Table 4 shows the difference in body weight perception by community and gender.  Table 5 shows the Feel minus Ideal Discrepancy of participants stratified by weight category. Underweight and normal-weight participants wanted to gain weight while overweight and obese participants desired to lose weight. There were no differences in FID by community and gender. Majority of participants were not doing anything to achieve their desired body image. Most participants attributed their current weight to hereditary (27.0%) and childbirth (27.0%) and also defined obesity as the accumulation of fat and a threat to health (91.0%) (Figures 2–5).

## 4. Discussion

This paper assessed the body image perceptions of rural and urban adults in the Ashanti region of Ghana. A prevalence of 52.8% was observed for overweight and obesity. Details of overweight and obesity prevalence and differences among rural and urban participants have been reported in a separate paper. The prevalence found in this study is within the range that has been reported by some studies conducted in Ghana. A systematic review by Ofori-Asenso pegged overweight and obesity prevalence in Ghana at 42.5% [23]. Participants who selected figures 3–9 as their CBI had median measured BMIs that closely matched the established BMI of the figures. Participants with median BMI of 20.5 and 21 chose figures 1 and 2, respectively. However, the established BMIs of figure 1 (18.3) and figure 2 (19.3) do not match the measured BMIs. This finding suggests the underestimation of weight among Ghanaians with normal BMI and the tendency to think and feel that they are underweight. This can lead to eating habits and practices that encourage weight gain and overweight. This also explains why normal-weight participants in this study, both males and females, desired to gain weight.

Male participants chose figure 5 as ideal for their gender while females chose figure 4 as ideal for their gender. For females, figure 4 was still chosen as their desired body image, but males chose a slightly lower figure (figure 4) as desired body image . Females also chose figure 5 as ideal for a male. Congruent with other studies, overweight was linked to wealth especially among rural participants. This may explain why a larger body image was taught to be ideal for males since they are expected to be financially stable in order to take care of their households [5, 8]. Cultural perceptions and ideals that link overweight and obesity to wealth and wellbeing undermine efforts to curb obesity and encourage preference for overweight and obesity. A systematic review of population-based studies has reported that rural areas in low- and middle-income countries drive 80% of BMI increases observed which calls for an integrated approach to dealing with rural malnutrition [24]. Selection of an overweight figure for a wealthy person may also be a result of study participants internalizing the situation in their communities. Among Africans, high socioeconomic status individuals have a higher prevalence of overweight and obesity [1]. The selection of a normal-weight figure by both gender is however an improvement in their weight perceptions as most studies have reported the desire for overweight and obese figures among Africans [5, 8]. This desire for a normal weight may be further explained by the view of obesity as a threat to health by almost all study participants. This improvement in body weight perception does not reflect on the current prevalence of overweight and obesity in the country and in Africa. This raises concerns about the current food environment which can make it difficult to achieve this desired normal body weight.

Half of the participants described obesity as the accumulation of fat while 39.5% viewed it as a mere increase in body weight. These findings suggest that Africans view overweight and obesity as a threat to health but have a distorted view of the condition and are unaware of the appropriate cutoffs and eventually underestimate their body size. Public health education, cultural-sensitive interventions, and screening for overweight and obesity can serve as means to correct this notion and make the population aware of their weight category.

Within the African context, most women after giving birth engage in eating habits and practices that promote excessive weight gain and this postpartum weight gain is viewed as an indicator of mother's wellbeing [25]. In rural areas especially, extended family support during the postpartum period renders mothers sedentary [26]. Majority of participants selected an overweight figure (figure 5) as ideal for a woman with children and figure 3 (normal weight) for a woman without children. This cut across for both males and females and for rural and urban participants. This indicates an association of overweight with childbirth. It is also a contributing factor to high prevalence of overweight and obesity observed among parous Ghanaian women [27]. In this study, participants attributed their current weight to childbirth and hereditary. It is possible for women to lose almost all the weight gained during pregnancy; therefore, childbirth should not be a hindrance to the attainment or maintenance of normal weight [28]. Antenatal and postnatal education should stress on the adaptation of healthy diet and increasing physical activity as means to lose weight after birth [29]. Intervention for new mothers at postnatal centres can be one of the sure ways of curbing the high prevalence of overweight and obesity among premenopausal African women.

Research suggests that individuals with genetic predisposition to obesity can adapt a healthy lifestyle to maintain an ideal body weight [30]. As it is said, the genes load the gun, but the environment pulls the trigger [31]. Additionally, excessive food intake rather than hereditary may be the contributing factor to obesity among participants. Members of a family tend to have similar eating patterns and may therefore have similar BMI ranges. Inappropriate perception of hereditary as a cause of obesity makes people give up on themselves and be reluctant to lose weight or implement weight loss strategies.

The World Health Organization recommends the use of appropriate diet and physical activity as a means to maintain a healthy weight [32]. It was observed from this study that most participants were not putting in efforts to achieve their desired body image. This may be because participants attributed their current weight to hereditary, hence were not willing to engage in any activity to obtain an ideal weight. Few participants engaged in either exercise or dieting to achieve their desired body weight. Fewer participants engaged in both exercise and dieting. Appropriate diet and exercise complement each another to ensure effective weight loss and maintenance of an ideal body weight. Use of diet alone as a means to lose weight may lead to restricted dietary intake and intake of nutrients below recommendations. This calls for public education on the importance of both diet and exercise as means to lose excess weight and maintain an ideal weight.

## 5. Conclusion

Findings from this study showed a preference for a body image within the normal range and an association of wealth and childbirth with overweight. Study participants viewed obesity as an accumulation of fat and a threat to their health. Participants attributed their current weight to hereditary or childbirth and most normal-weight participants wanted to gain weight. Rural participants selected a significantly higher figure for a wealthy person and women selected a higher figure as ideal body weight and ideal weight for the opposite sex. These findings when compared with earlier studies show an improvement in body weight and obesity perception. There is however the need for public education, individual empowerment, frequent screening for overweight and obesity, creation of supportive food environments, and an adaptation of a multifaceted approach and cultural-sensitive interventions as a means to control obesity.

## Figures and Tables

**Table 1:** Current body weight perception by actual BMI.

Stunkard images	Corresponding BMI	Number of participants choosing image as current weight		BMI median (IQR)^∗^		Number of participants choosing image as current weight		BMI median (IQR)^∗^	
Figure number		Rural, *n* (%)	Urban, *n* (%)	Rural	Urban	Male, *n* (%)	Female, *n* (%)	Male	Female
1	18.3	10 (3.5)	11 (4.1)	20.2 (3.4)	20.7 (4.5)	3 (2.7)	18 (4.0)	19.5 (0)	20.5 (4.5)
2	19.3	30 (10.4)	23 (8.6)	20.9 (2.6)	21.6 (4.1)	9 (8.0)	44 (9.9)	20.5 (4.0)	21.1 (3.6)
3	20.9	51 (17.6)	30 (11.2)	22.6 (3.1)	21.9 (3.8)	14 (12.5)	68 (15.3)	20.7 (3.7)	22.5 (3.3)
4	23.1	54 (18.7)	62 (23.1)	24.2 (3.9)	25.0 (5.0)	27 (24.1)	88 (19.8)	23.0 (2.7)	25.2 (4.7)
5	26.2	68 (23.5)	62 (23.1)	26.0 (5.8)	26.8 (5.9)	32 (28.6)	98 (22.0)	23.2 (2.9)	27.7 (5.1)
6	29.9	41 (14.2)	46 (17.2)	27.9 (5.9)	30.4 (4.3)	13 (11.6)	74 (16.6)	26.3 (2.6)	30.1 (5.8)
7	34.3	29 (10.0)	21 (7.8)	31.5 (6.9)	34.0 (8.1)	11 (9.8)	39 (8.8)	32.7 (7.3)	32.6 (8.8)
8	38.6	41 (1.4)	12 (4.5)	35.7 (11.3)	40.3 (7.3)	3 (2.7)	13 (2.9)	30.2 (0)	39.3 (7.2)
9	45.4	2 (0.7)	1 (0.4)	42.4 (0)	55.5 (0)	0 (0)	3 (0.7)	0 (0)	46.5 (0)

Corresponding BMI refers to the known BMI of the Stunkard figure. ^*∗*^Actual measured BMI of study participants.

**Table 2:** Body weight perception of male participants.

Stunkard images	Corresponding BMI	Desired body weight	IBW for gender	Unhealthiest image for gender	IBW for opposite sex	Wealthy man	Wealthy woman	Woman with children no more reproducing	Woman with children still reproducing	Woman without children
Figure		*n* (%)	*n* (%)	*n* (%)	*n* (%)	*n* (%)	*n* (%)	*n* (%)	*n* (%)	*n* (%)
1	18.3	3 (2.7)	3 (2.7)	10 (9.0)	4 (3.5)	2 (1.8)	2 (1.8)	0 (0)	3 (2.7)	20 (18.0)
2	19.3	5 (4.4)	6 (5.5)	0 (0)	7 (6.2)	1 (0.9)	1 (0.9)	4 (3.7)	22 (19.6)	24 (21.6)
3	20.9	12 (10.6)	9 (8.2)	1 (0.9)	20 (17.7)	2 (1.8)	5 (4.5)	10 (9.3)	30 (26.8)	31 (27.9)
4	23.1	42 (37.2)	38 (34.5)	2 (1.8)	47 (41.6)	8 (7.1)	17 (15.5)	29 (26.9)	34 (30.4)	26 (23.4)
5	26.2	33 (29.2)	46 (41.8)	0 (0)	30 (26.5)	33 (29.5)	33 (30.0)	29 (26.9)	15 (13.4)	7 (6.3)
6	29.9	15 (13.3)	6 (5.8)	0 (0)	5 (4.4)	30 (26.8)	28 (25.5)	17 (15.7)	6 (5.4)	3 (2.7)
7	34.3	2 (1.8)	2 (1.8)	4 (3.6)	0 (0)	20 (17.9)	9 (8.2)	10 (9.3)	1 (0.9)	0 (0)
8	38.6	0 (0)	0 (0)	19 (17.1)	0 (0)	10 (8.9)	11 (10.0)	3 (2.8)	1 (0.9)	0 (0)
9	45.4	1 (0.9)	0 (0)	77 (67.6)	0 (0)	6 (5.4)	4 (3.6)	6 (5.6)	0 (0)	0 (0)

Perceptions of weight are presented in frequencies and percentages.

**Table 3:** Body weight perceptions of female participants.

Stunkard images	Corresponding BMI	Desired body weight	IBW for gender	Unhealthiest image for gender	IBW for opposite sex	Wealthy man	Wealthy woman	Woman with children no more reproducing	Woman with children still reproducing	Woman without children
Figure		*n* (%)	*n* (%)	*n* (%)	*n* (%)	*n* (%)	*n* (%)	*n* (%)	*n* (%)	*n* (%)
1	18.3	18 (4.0)	13 (2.9)	48 (10.9)	17 (3.8)	3 (0.7)	6 (1.4)	9 (2.1)	28 (6.5)	67 (15.5)
2	19.3	63 (14.1)	42 (9.5)	2 (0.5)	26 (5.9)	12 (2.8)	9 (2.1)	22 (5.2)	64 (14.8)	80 (18.5)
3	20.9	109 (24.3)	101 (22.9)	3 (0.7)	58 (13.1)	19 (4.4)	31 (7.2)	61 (14.4)	126 (29.1)	121 (28)
4	23.1	111 (24.6)	121 (27.4)	2 (0.5)	102 (23.0)	38 (8.8)	54 (12.6)	87 (20.5)	107 (24.7)	72 (16.7)
5	26.2	94 (20.8)	116 (26.3)	1 (0.2)	155 (35.0)	77 (17.9)	114 (26.6)	109 (25.6)	63 (14.5)	46 (10.6)
6	29.9	33 (7.3)	33 (7.5)	5 (1.1)	71 (16.0)	116 (26.9)	98 (22.8)	72 (16.9)	34 (7.9)	30 (6.9)
7	34.3	15 (3.3)	11 (2.5)	8 (1.8)	5 (1.1)	62 (14.4)	51 (11.9)	30 (7.1)	5 (1.2)	9 (2.1)
8	38.6	5 (1.1)	2 (0.5)	56 (12.7)	5 (1.1)	50 (11.6)	34 (7.9)	12 (2.8)	4 (0.9)	1 (0.2)
9	45.4	0 (0)	2 (0.5)	315 (71.6)	4 (0.9)	54 (12.5)	32 (7.5)	23 (5.4)	2 (0.5)	6 (1.3)

**Table 4:** Differences by community and gender on body weight perceptions.

Perceptions of weight	Rural	Urban	*p* value	Male	Female	*p* value
	Median (IQR)	Median (IQR)		Median (IQR)	Median (IQR)	
Current weight	5 (3)	5 (2)	0.205	5 (2)	5 (3)	0.763
Desired weight	4 (2)	4 (2)	0.323	4 (1)	4 (2)	<0.001^*∗*^
Ideal weight for gender	4 (2)	4 (2)	0.578	4 (1)	4 (2)	0.008^*∗*^
Unhealthiest	9 (1)	9 (0.25)	0.021^*∗*^	9 (1)	9 (1)	0.543
Ideal for opposite sex	4 (1)	5 (1)	0.435	4 (2)	5 (1)	<0.001^*∗*^
Wealthy man	6 (3)	6 (2)	<0.001^*∗*^	6 (2)	6 (2)	0.184
Wealthy woman	6 (2)	5 (2)	<0.001^*∗*^	5 (1)	6 (2)	0.590
Woman with children no more reproducing	5 (2)	5 (2)	0.841	5 (2)	5 (2)	0.328
Woman with children still reproducing	4 (2)	3 (1)	0.242	4 (1)	4 (2)	0.796
Woman without children	3 (2)	3 (2)	0.075	3 (2)	3 (2)	0.066

Median figure chosen for different perception responses.

**Table 5:** Body satisfaction by weight groups.

Actual weight category	Feel minus Ideal Discrepancy					
	Male, mean ± SD	Female, mean ± SD	*p* value	Urban, mean ± SD	Rural, mean ± SD	*p* value
Underweight	0^*∗*^	−1.0 ± 1.1	—	−1.2 ± 1.3	−0.8 ± 0.9	0.570
Normal	−0.32 ± 1.3	−0.33 ± 1.2	0.963	−0.42 ± 1.3	−0.2 ± 1.2	0.190
Overweight	0.8 ± 1.6	0.7 ± 1.3	0.860	0.8 ± 1.3	0.7 ± 1.5	0.678
Obese	2.0 ± 1.0	2.0 ± 1.4	1.00	1.9 ± 1.6	2.0 ± 1.3	0.904

Feel minus ideal discrepancy was calculated by subtracting the current body image from the desired body image.

## Data Availability

The data analyzed for this manuscript are available from the corresponding author and can be made accessible upon reasonable request.

## References

[1] Agyemang C., Boatemaa S., Agyemang Frempong G., de-Graft Aikins A. (2016). Obesity in sub-Saharan Africa. *Metabolic Syndrome*.

[2] Hoffman J. A., Rosenfeld L., Schmidt N. (2015). Implementation of competitive food and beverage standards in a sample of Massachusetts schools: the NOURISH Study (nutrition opportunities to understand reforms involving student health). *Journal of the Academy of Nutrition and Dietetics*.

[3] Igumbor E. U., Sanders D., Puoane T. R. (2012). “Big food,” the consumer food environment, health, and the policy response in South Africa. *PLoS Medicine*.

[4] Samdal G. B., Eide G. E., Barth T., Williams G., Meland E. (2017). Effective behaviour change techniques for physical activity and healthy eating in overweight and obese adults; systematic review and meta-regression analyses. *International Journal of Behavioral Nutrition and Physical Activity*.

[5] Appiah C. A., Otoo G. E., Steiner-Asiedu M. (2016). Preferred body size in urban Ghanaian women: implication on the overweight/obesity problem. *Pan African Medical Journal*.

[6] Duncan D. T., Wolin K. Y., Scharoun-Lee M., Ding E. L., Warner E. T., Bennett G. G. (2011). Does perception equal reality? Weight misperception in relation to weight-related attitudes and behaviors among overweight and obese US adults. *International Journal of Behavioral Nutrition and Physical Activity*.

[7] Lynch E., Liu K., Wei G. S., Spring B., Kiefe C., Greenland P. (2009). The relation between body size perception and change in body mass index over 13 years: the coronary artery risk development in young adults (CARDIA) study. *American Journal of Epidemiology*.

[8] Holdsworth M., Gartner A., Landais E., Maire B., Delpeuch F. (2004). Perceptions of healthy and desirable body size in urban Senegalese women. *International Journal of Obesity*.

[9] Puoane T., Fourie J. M., Shapiro M., Rosling L., Tshaka N. C., Oelefse A. (2005). Big is beautiful—an exploration with urban black community health workers in a South African township. *South African Journal of Clinical Nutrition*.

[10] Flegal K. M., Kit B. K., Orpana H., Graubard B. I. (2013). Association of all-cause mortality with overweight and obesity using standard body mass index categories. *JAMA*.

[11] Popkin B. M., Adair L. S., Ng S. W. (2012). Global nutrition transition and the pandemic of obesity in developing countries. *Nutrition Reviews*.

[12] Giskes K. (2016). Policy responses and obesogenic food environments. *Geographies of Obesity: Environmental Understandings of the Obesity Epidemic*.

[13] Chintu C., Mwinga A. (1999). An African perspective on the threat of tuberculosis and HIV/AIDS-can despair be turned to hope?. *The Lancet*.

[14] Renzaho A. M. N., McCabe M., Swinburn B. (2012). Intergenerational differences in food, physical activity, and body size perceptions among African migrants. *Qualitative Health Research*.

[15] Toselli S., Rinaldo N., Gualdi-Russo E. (2016). Body image perception of African immigrants in Europe. *Globalization and Health*.

[16] Okop K. J., Mukumbang F. C., Mathole T., Levitt N., Puoane T. (2016). Perceptions of body size, obesity threat and the willingness to lose weight among black South African adults: a qualitative study. *BMC Public Health*.

[17] Okop K. J., Levitt N., Puoane T. (2019). Weight underestimation and body size dissatisfaction among black African adults with obesity: implications for health promotion. *African Journal of Primary Health Care & Family Medicine*.

[18] Matoti-Mvalo T., Puoane T. (2011). Perceptions of body size and its association with HIV/AIDS. *South African Journal of Clinical Nutrition*.

[19] Benkeser R. M., Biritwum R., Hill A. G. (2012). Prevalence of overweight and obesity and perception of healthy and desirable body size in urban, Ghanaian women. *Ghana Medical Journal*.

[20] Mogre V., Aleyira S., Nyaba R. (2015). Misperception of weight status and associated factors among undergraduate students. *Obesity Research & Clinical Practice*.

[21] Patt M. R., Lane A. E., Finney C. P., Yanek L. R., Becker D. M. (2002). Body image assessment: comparison of figure rating scales among urban Black women. *Ethnicity & Disease*.

[22] Stunkard A. J., Sørensen T., Schulsinger F. (1983). Use of the Danish Adoption Register for the study of obesity and thinness. *Research Publications—Association for Research in Nervous and Mental Disease*.

[23] Ofori-Asenso R., Agyeman A. A., Laar A., Boateng D. (2016). Overweight and obesity epidemic in Ghana—a systematic review and meta-analysis. *BMC Public Health*.

[24] NRF Collaboration (2019). Rising rural body-mass index is the main driver of the global obesity epidemic in adults. *Nature*.

[25] Dake F. A., Tawiah E. O., Badasu D. M. (2011). Sociodemographic correlates of obesity among Ghanaian women. *Public Health Nutrition*.

[26] de-Graft Aikins A. (2014). Food beliefs and practices during pregnancy in Ghana: implications for maternal health interventions. *Health Care for Women International*.

[27] Tuoyire D. A., Kumi-Kyereme A., Doku D. T. (2016). Socio-demographic trends in overweight and obesity among parous and nulliparous women in Ghana. *BMC Obesity*.

[28] Ketterl T. G., Dundas N. J., Roncaioli S. A., Littman A. J., Phipps A. I. (2018). Association of pre-pregnancy BMI and postpartum weight retention before second pregnancy, Washington state, 2003–2013. *Maternal and Child Health Journal*.

[29] Liu J., Wilcox S., Whitaker K., Blake C., Addy C. (2015). Preventing excessive weight gain during pregnancy and promoting postpartum weight loss: a pilot lifestyle intervention for overweight and obese African American women. *Maternal and Child Health Journal*.

[30] Temelkova-Kurktschiev T., Stefanov T. (2012). Lifestyle and genetics in obesity and type 2 diabetes. *Experimental and Clinical Endocrinology & Diabetes*.

[31] Saey T. H. (2014). Humans and society: mummies reveal hardened arteries: pharaohs, ancient Peruvians, many others had heart disease. *Science News*.

[32] Mendis S., Puska P., Norrving B., Organization W. H. (2011). *Global Atlas on Cardiovascular Disease Prevention and Control*.

